# ASCOT analysis with atorvastatin shows limits of CRP as indicator of cardiovascular risk

**Published:** 2011

The addition of high-sensitivity C-reactive protein (hs-CRP) measurements did not much improve conventional risk assessments in patients with hypertension and other cardiovascular (CV) risk factors in a post hoc analysis of an Anglo– Scandinavian Cardiac Outcomes Trial (ASCOT) lipid-lowering arm.^1^

The lipid-lowering arm of ASCOT, which involved more than 10 000 patients, showed that atorvastatin therapy achieved significant reduction in major cardiovascular events in hypertensive patients with normal or modestly elevated low-density lipoprotein cholesterol (LDL-C) levels. Also, the combination of atorvastatin/ amlodipine as analysed in both the blood pressure-lowering and lipid arms of the ASCOT trial showed a greater reduction in non-fatal myocardial infarction (MI) and fatal coronary artery disease compared to patients receiving the atenolol- based therapy.

This post hoc ASCOT assessment of the value of hs-CRP was based on data from 485 patients from the UK and Irish centres who reached a composite endpoint that included CV death, nonfatal MI, coronary revascularisation, or fatal/non-fatal stroke, and who were age and gender matched to 1 367 patients in the control group who hadn’t suffered one of those events. They were followed for 5.5 years.

Baseline levels of hs-CRP and LDL-C were significantly correlated with each other at p < 0.0001, and both parameters were significantly predictive of a composite CV endpoint that included CV death, non-fatal MI, coronary revascularisation, or fatal/non-fatal stroke, at an odds ratio (OR) of 1.31 (95% CI: 1.10–1.56, p = 0.002) for LDL-C and OR 1.19 (95% CI: 1.05–1.34, p = 0.006) for every one standard deviation increase in hs-CRP.

According to Dr Peter Sever, Imperial College, London, including hs-CRP into modified and full Framingham risk models only minimally improved prediction of cardiovascular events, while the addition of on-treatment hs-CRP measurement to on-treatment LDL-C measurement did not improve the prediction of patient response to the statin therapy.

Also, among patients assigned to atorvastatin:

- Baseline hs-CRP did not predict the magnitude of the drug’s effect on cardiovascular endpoints.
- Over six months of atorvastatin therapy, the median LDL-C fell by 40.3% and the median hs-CRP dropped by 27.4%.

Lower on-treatment LDL-C but not hs-CRP was associated with a highly significant reduction in cardiovascular events over six months:

- An LDL-C less than the median of 2.1 mmol/l, compared with LDL-C at or higher than the median predicted a reduction in the composite endpoint at an adjusted OR of 0.41 (95% CI: 0.22–0.75, p = 0.004).
- But an hs-CRP less than the median of 1.83 mg/l, compared to hs-CRP at or higher than the median, showed no such predictive effect at an adjusted OR of 0.86 (95% CI: 0.49–1.51, p = 0.60).

‘It is clear that achievement of low LDL cholesterol levels confers great benefit, and there’s no added benefit by achieving a lower CRP level’, Sever said in his presentation. Among patients in the analysis who had been randomised to receive atorvastatin, a significant reduction in LDL-C corresponded to a significant drop in CV event risk at six months. But a significant fall in hs-CRP levels did not predict a decrease in CV events.

‘These results do not support current proposals to measure CRP in the clinical setting, either to assign statins to individuals on the basis of an elevated CRP alone or to monitor CRP levels as an indicator of the efficacy of statin treatment’, he said.

The ASCOT analysis, according to Sever, challenges the recent broadening of rosuvastatin indications by the US FDA, based on the JUPITER trial, which compared the drug with placebo in more than 17 000 adults with normal LDL-C and hs-CRP ≥ 2.0 mg/l.^2^

Sever also pointed out that in the ASCOT analysis, adding CRP to a conventional risk assessment based on elements of the Framingham risk score improved risk prediction, but not by much. ‘If you measure their total cholesterol-to-HDL cholesterol ratio, if you measure their blood pressure, if you’ve decided whether or not they smoke, [and consider] their age and their sex, that tells you all you need to know about their risk’, he said. ‘Putting CRP in there doesn’t alter the [risk-status] classification of people, and that suggests to me that it doesn’t have added value.’

That means CRP does not have a practical role in deciding whether to initiate statin therapy, according to Sever. ‘In patients with hypertension, they’re on statins. In patients with diabetes, they’re on statins. In patients with stable coronary artery disease, they’re on statins. There’s no additional benefit to be gained from measuring CRP.’

Discussing the ASCOT analysis, Dr Elliot Antman (Brigham and Women’s Hospital) seemed to agree that CRP probably has a limited risk-stratification role. ‘For patients with a very low risk-factor profile [based on standard risk assessment], a measurement of CRP might be considered as a sort of general screen’, he said. ‘But I’m not so convinced that it’s going to provide a lot of incremental value for deciding who should start on a statin.’

‘If all one has to go on is CRP level, there’s evidence that it can be predictive’, Antman observed. ‘But if you start giving me incremental information like this person smokes, or has diabetes, or has hypertension, then the amount of benefit I’m gaining from knowing the person’s CRP is dropping with each new piece of information you give me.’
